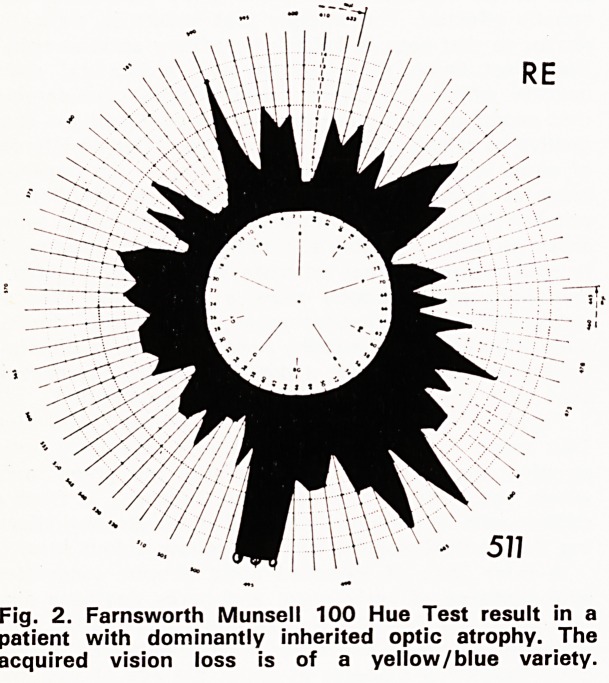# The Hereditary Optic Atrophies

**Published:** 1975-04

**Authors:** W. S. Foulds

**Affiliations:** Tennent Institute of Ophthalmology, University of Glasgow


### The Hereditary Optic Atrophies

By

**Professor W. S. Foulds, M.D., Ch.M., F.R.C.S.**

Tennent Institute of Ophthalmology, University of Glasgow

There are a number of hereditary conditions in which optic atrophy may be a feature. These include skeletal conditions such as cranio-stenosis, some of the phakomatoses, congenital glaucoma and so on. In these conditions the optic atrophy is the result of a fairly obvious mechanism such as optic nerve compression, the effects of intraocular pressure on circulation in the nerve head or optic atrophy secondary to retinal damage such as is seen in Leber's hereditary amaurosis. I am not, however, going to consider this group further, restricting my talk to consideration of *Leber's hereditary optic atrophy* and to *dominantly inherited optic atrophy.*

As you know, Leber's hereditary optic atrophy is a bilateral condition affecting males more often than females. It is characterised by a sudden onset of optic neuritis with papillitis in each eye, going on to bilateral primary optic atrophy with permanently poor central vision. The onset usually occurs in the teens but may be in childhood or in adult life. Although both eyes are affected, there is usually some asymmetry about the time of onset, and sometimes there may be a gap of weeks before the second eye is affected. In most cases a slight spontaneous improvement in vision occurs some months after the onset. The final acuity, however, is commonly in the region of 3/60 with a dense permanent central scotoma. It has been noted by some authors that a small proportion, somewhere between one and four per cent, of cases of Leber's hereditary optic atrophy may recover useful vision spontaneously.

The inheritance of Leber's hereditary optic atrophy is in dispute. It bears some of the hallmarks of a sex-linked recessive mode of inheritance, but the number of cases occurring does not fit the expected incidence of the condition. It is for this reason, among others, that it has been suggested that the inherited defect is a biochemical one and is only made manifest when certain other non-genetic factors operate. It is certainly true that, even in one family, the age of onset of the acute episode may be very variable and this variability in the pattern of onset does suggest a multifactorial aetiology in which a genetic factor is only one of the factors involved. As an alternative to a genetic inheritance, it has been suggested that the atypical pattern of inheritance could be explained on the basis of cytoplasmic transmission, it being postulated, for example, that the condition might be a virus infection of the cytoplasm of the ovum and not necessarily a defect carried on the chromosomal apparatus.

In practice, we do not diagnose Leber's hereditary optic atrophy unless there is one other maternally related member of the family affected. Sometimes, however, it is difficult to establish a family history. On one occasion, we had two suspects in the ward, each denying that they had a family history of the disease. Subsequently, we found out that, unknown to either, they shared the same grandmother through the maternal line.

As far as dominantly inherited optic atrophy is concerned, it is less common than Leber's atrophy and less well recognised. Its onset is similar to Leber's atrophy but tends to be at a younger age. We have seen cases starting at ages ranging from two years to fourteen years. The inheritance of this condition is that of an autosomal dominant and the sexes are equally affected. The atrophy that occurs is clinically similar to that seen in Leber's hereditary optic atrophy, the onset being fairly abrupt with a bilateral optic neuritis which sometimes presents with considerable disc swelling, even masquerading as papilloedema suggestive of raised intracranial pressure. After an interval of some weeks the swelling gives way to the development of atrophy and the final picture is of bilateral primary optic atrophy with a visual defect of the same order as seen in Leber's hereditary optic atrophy. It is said, however, that whereas patients with Leber's hereditary optic atrophy show a preferential loss of red/green colour discrimination, those with dominantly inherited optic atrophy have difficulty in discriminating yellow and blue, a defect more usually associated with a retinal than an optic nerve condition.

Figure 1 shows the Farnsworth Munsell 100 Hue Test result in a patient with Leber's hereditary optic atrophy showing the characteristic red/green axis which one finds in this condition. Figure 2 is from a patient with dominantly inherited optic atrophy showing the vertical polarity of blue/yellow colour loss.

A rarer form of inherited optic atrophy associated with diabetes of early onset and nerve deafness also occurs. In one family we have under observation, two members, a brother and sister, who have been affected. Each developed diabetes at about the age of two and optic atrophy and deafness commenced at about the age of ten. The sister is some ten years older than her brother, and by the late teens the atrophy had proceeded to complete blindness in both eyes and the nerve deafness was also severe. In the brother, when we saw him, there was a moderate reduction of acuity to 6/12 or 6/18 accompanied by a loss of red/green colour discrimination similar to that seen in Leber's atrophy.

Interest in the possible therapy of these conditions

**Fig. 1. Farnsworth Munsell 100 Hue Test result in a patient with Leber's hereditary optic atrophy. The acquired colour vision loss is of a red/green variety.**

**Fig. 2. Farnsworth Munsell 100 Hue Test result in a patient with dominantly inherited optic atrophy. The acquired vision loss is of a yellow/blue variety.**

was aroused by the suggestion that, biochemically, Leber's hereditary optic atrophy might be related to some of the toxic optic neuropathies and, in particular, to tobacco amblyopia, a condition which is known to be reversible (Wilson, 1963). I think it would be useful at this point to say a little of our current beliefs about tobacco amblyopia, a condition in which I have been interested for some years and which presents many fascinating biochemical features.

We know that tobacco amblyopia is a disease affecting a small proportion of pipe-smoking elderly males, although it does, however, occur more rarely in younger patients, in females and in those taking tobacco as cigarettes, cigars, snuff, and so on.

We know that cessation of smoking leads to recovery of vision in three to six months, as does treatment with $B^{12}$ given intramuscularly as hydroxocobalamin, although not as cyanocobalamin (Chisholm et al, 1967).

We also know that giving sulphur in a suitable form may lead to recovery in this condition. We, ourselves, have successfully treated tobacco amblyopia with the sulphur-containing amino acid cystine while others have claimed success for the administration of thiosulphate (Phillips et al, 1970).

Whichever method of treatment is chosen, we find that the same biochemical changes occur, namely, an increase in the plasma level of thiocyanate and an increased excretion of this substance in the urine coupled with an increased renal clearance of thiocyanate (Chisholm and Pettigrew, 1970).

Other factors of importance in tobacco amblyopia are that about 40% of cases show overt or latent evidence of $B^{12}$ deficiency. Fifty per cent or so can be shown to take a diet deficient in protein (Foulds et al, 1969) while most patients with tobacco amblyopia show an abnormality of sulphur metabolism characterised by deficient red cell glutathione in the blood (Pettigrew et al, 1972). This is an important source of sulphydryl groups and its concentration in the red cells is known to be depressed in patients with pernicious anaemia. We have found that glutathione levels in the red cells of patients with tobacco amblyopia are low even where there is no evidence of $B^{12}$ deficiency. Can we put all these facts together.

Wokes (1958) first suggested that the toxic factor in tobacco causing amblyopia, might be cyanide and this hypothesis receives support from some of our biochemical results. It is, however, our belief that tobacco amblyopia is primarily a defect of sulphur metabolism brought on by either a deficient intake of sulphur-containing amino acids in the diet such as occurs when a low protein diet is taken or by a failure to elaborate certain sulphur-containing amino acids which require $B^{12}$ for their manufacture. The latter group comprises those with pernicious anaemia, certain types of abnormality of intestinal absorption of those lacking vitamin $B^{12}$ in their diet. Many patients with tobacco amblyopia take a diet which is both poor in protein and in $B^{12}$ and both factors combine to depress the levels of sulphur-containing amino acids in the blood and tissues. Apart from dietary lack and poor $B^{12}$ absorption, some other unrecognised defect in the metabolism of sulphur may be present in those patients with tobacco amblyopia who cannot be demonstrated as being $B^{12}$ deficient or protein deficient but who, nevertheless, show abnormally low levels of glutathione in the blood.

Sulphur is necessary to detoxify cyanide to its non-toxic metabolite thiocyanate and it is our postulate that lack of a suitable sulphur donor for this purpose is the defect in tobacco amblyopia.

It is probably worth stressing again that in patients who smoke heavily but who do not have tobacco amblyopia, there is an elevated thiocyanate level in the plasma and urine indicating the normal detoxification of cyanide by conjugation to sulphur. Cessation of smoking is accompanied by a fall in the thiocyanate level because the cyanide intake has been reduced. This is quite different to the situation in tobacco amblyopia and, indeed, in Leber's hereditary optic atrophy where thiocyanate levels are persistently low even when it is known that there is a high intake of cyanide. This suggests that, in these patients, there is an inability to detoxify cyanide to thiocyanate.

It was at one time suggested that hydroxocobalamin might detoxify cyanide directly by converting it to cyanocobalamin (Smith, 1961). We think this is unlikely for if this detoxification route played a significant role, then one would expect cyanide to be excreted as cyanocobalamin and the level of thiocyanate in the blood and urine to fall or be unaltered. The rise in thiocyanate concentration which follows the administration of hydroxocobalamin may be explained by considering the role that co-enzyme $B^{12}$ has in the elaboration of cystine, cysteine and methionine and, as we think, presenting sulphur in a suitable form for conjugation with cyanide.

Additional evidence that this occurs is the increase in red cell glutathione which follows the giving of hydroxocobalamin. Treating patients with tobacco amblyopia with cyanocobalamin instead of hydroxocobalamin may merely add further cyanide to the cyanide pool and, certainly, in experimental animals, we have shown that labelled carbon from the cyanide radical given parenterally as cyanocobalamin accumulates preferentially in the optic nerve as compared with other tissues (Foulds et al, 1972).

What parallels can one draw between tobacco amblyopia and Leber's hereditary optic atrophy? Biochemically, the parallels are definite for in both the thiocyanate levels in blood and urine are lower than expected and in both these levels rise to normal values following the administration of either hydroxocobalamin or cystine.

Red cell glutathione levels too are pathologically low in Leber's hereditary optic atrophy and again rise to near normal levels on treatment with hydroxocobalamin or cystine. Attempts to estimate actual cyanide levels in these conditions in the past have been thwarted by the difficulty of identifying such small quantities of such a labile radical. We have found, for example, that estimations of plasma levels of cyanide depend greatly on how long the plasma remains in contact with red cells after the specimen is withdrawn, for cyanide progressively enters the red blood cells from the plasma (Pettigrew and Fell, 1973). We now have methods for the estimation of cyanide in whole blood but, so far, these do not show excess cyanide in the blood of patients with either tobacco amblyopia or Leber's hereditary optic atrophy. Blood levels may, however, be no guide to tissue levels and, at present, we are forced to use measurements of thiocyanate in our assessment of cyanide load and its detoxification.

In other respects Leber's hereditary optic atrophy and tobacco amblyopia are rather different, thus the field defect in the two conditions is dissimilar, tobacco amblyopia showing a relative depression in the central-caecal area while Leber's hereditary optic atrophy shows an extensive central scotoma which usually includes the blind spot.

As Leber's hereditary optic atrophy develops, however, it does go through a phase of centro-caecal depression and it may be that the defect in Leber's hereditary optic atrophy is merely quantitatively different rather than qualitatively so.

When tobacco amblyopia has been present for a long time, that is, in excess of a year, then primary optic atrophy develops and, when this occurs, a permanent defect of vision is invariable.

What about the response to therapy? We know that the loss of vision in tobacco amblyopia is reversible provided the condition has not been present for too long and, even then, a significant improvement in vision is likely within six months if smoking is stopped or if the patient is given large doses of intra-muscular hydroxocobalamin or large oral dosage with the amino acid, cystine. Obviously, one is interested in whether the visual loss in Leber's hereditary optic atrophy is similarly reversible. It is very difficult to reach conclusions about the value of therapy in Leber's atrophy, partly because of the variability of the natural history of the disease and partly because it is difficult to find a sufficient number of cases of recent onset to treat. So far, we have treated between 25 and 30 cases of Leber's hereditary optic atrophy with the same regimen that we have used for tobacco amblyopia. The majority of these patients had well established primary optic atrophy and, as might be expected, in these cases where the condition had been present for more than three years, no significant change in vision occurred following treatment, although the biochemical changes I have mentioned occurred in practically all. With cases of more recent onset, the picture is less clear. So far we have treated 12 cases where the onset of visual loss has been of three years duration or less and 6 cases where the visual loss has been of one year's duration or less. In this group of 6 patients, 2 have recovered normal central acuity, one patient to 6/5 and one to 6/6. A third patient has shown a useful improvement from counting fingers to 6/36. Of the three remaining patients in this group, the vision in one patient remained unaltered, while in the other two, although central vision is, as yet, poor, the visual field has shown considerable improvement. Treatment in these cases is still continuing and we are hopeful that an improvement in the visual acuity will follow.

We are well aware that, because of the occasional occurrence of spontaneous improvement in this condition, no firm conclusion with regard to the efficacy of treatment can be drawn as yet, but the apparent difference in these groups at least prompts us to continue investigation of the effects of therapy in this condition. It is interesting that the patients recovering normal vision continued to improve during two years of therapy, and that in both cases little change occurred until treatment had been used for a year. In each case, the improvement in visual acuity was preceded by an improvement in visual field and it is this which makes up hope that visual improvement will still occur in some of our cases of more recent onset who have been treated for a year or less.

We do not think this is a condition which lends itself to a controlled trial, but if therapy is effective in cases of recent onset, it should not be too difficult to show that these patients behave differently as a group from the main bulk of untreated patients.

In terms of biochemical normalisation, it is our impression that oral cystine combined with hydroxocobalamin may be better than either alone in the treatment of Leber's optic atrophy and our current regimen is to give these patients 8 gms. of cystine orally per day combined with 1,000 micrograms of hydroxocobalamin three times weekly. Treatment is continued for two years and for longer if improvement is occurring.

We are unable to offer any explanation for the slow recovery of vision when it occurs or whether this is spontaneous or as the result of therapy. It may be that some slow reparative process, such as remyelination, is occurring and whether therapy is contributing directly to this or merely preventing further damage and so allowing normal reparative processes to operate, is impossible to say.

As regards therapy in dominantly inherited optic atrophy, we have no evidence that the same mechanism as may be operative in Leber's atrophy is present in dominantly inherited atrophy. Thus serum thiocyanate levels among those patients resemble normal smokers and non-smokers and are not depressed. We have seen nine patients from five families with this type of atrophy and treated eight of them with hydroxocobalamin without any improvement in vision. At present, not only are we unaware of the mechanism underlying this defect, we do not really know whether the defect is in the optic nerve or in the neuro-sensory retina. Electrophysiological tests and the optic atrophy which occurs would seem to implicate the optic nerve. Tests of colour vision are suggestive of retinal rather than optic nerve damage.

As regards one recent patient we have seen with familial optic atrophy complicating early onset diabetes which was also familial, this boy has been treated with hydroxocobalamin and cystine. His sister developed diabetes at the age of three. By the age of nine, the visual acuity was 6/24, by the age of eleven 6/60, by the age of thirteen 2/60, by the age of fifteen—no perception of light. The brother also developed diabetes at the age of three. At the age of eleven his visual acuity was 6/9, at the age of fourteen 6/12, when he was noted to have pallor of the optic discs, a red/green colour defect and visual field loss. Treatment was commenced and has been continued for two years. He is now aged eighteen and visual acuity is 6/18. We are quite unable to say, at present, whether treatment has modified the rate of deterioration in this child.

### REFERENCES

CHISHOLM, I. A., Bronte-Stewart, J. M. and Foulds, W.S. (1967). Lancet, **ii**, 450-451. Hydroxocobalamin versus cyanocobalamin in the treatment of tobacco amblyopia.

CHISHOLM, I. A. and Pettigrew, A. R. (1970) Trans. Ophthal. Soc. U.K., **90**, 827-38. Biochemical observations in toxic optic neuropathy.

FOULDS, W. S., Chisholm, I. A., Bronte-Stewart, J. M. and Wilson, T. M. (1969). Brit. J. Ophthal., **53**, 393-7. Vitamin $B_{12}$ absorption in tobacco amblyopia.

FOULDS, W. S., Chisholm, I. A. and Miller, W. T. (1972) The Optic Nerve, Proceedings of Second Mackenzie Memorial Symposium, Ed. J. S. Cant, Kimpton, London, p. 270.

PETTIGREW, A. R., Fell, G. S. and Chisholm, I. A. (1972) Exp. Eye Res., **14**, 87-90 Sept. Red cell glutathione tobacco amblyopia.

PETTIGREW, A. R. and Fell, G. S. (1973) Clin. Chem., **19**, 466-71 May. Microdiffusion method for estimation of cyanide in whole blood and its application to the study of conversion of cyanide to thiocyanate.

PHILLIPS, C. I., Wang, M. K. and Van Peborgh, P. F. (1970) Trans. Ophthal. Soc. U.K., **90**, 809-26. Some observations on the mechanisms of tobacco amblyopia and its treatment with sodium thiosulphate.

SMITH, A. D. M. (1961) Lancet **i**, 1001-1002. Retrobulbar neuritis in addisonian pernicious anaemia.

WILSON, J. (1963) Brain, **86**, 347-362. Leber's Hereditary Optic Atrophy. Some Clinical and Aetiological Considerations.

WOKES, F. (1958) Lancet, **ii**, 668. Tobacco amblyopia.

## Figures and Tables

**Fig. 1. f1:**
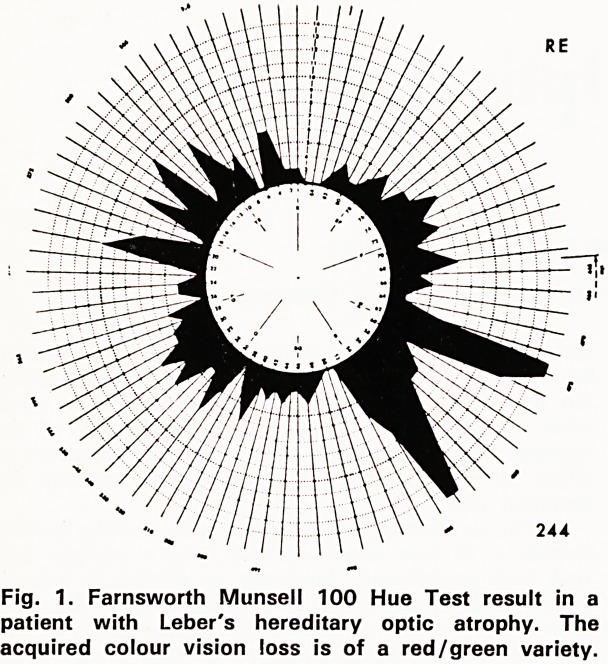


**Fig. 2. f2:**